# New approaches in the analysis of spent embryo culture media in the IVF process

**DOI:** 10.1007/s00404-025-08017-3

**Published:** 2025-04-28

**Authors:** Zuzana Badovská, Katarína Dubayová, Lukáš Smolko, Silvia Toporcerová, Ivana Lukáčová, Dominika Šeršeň, Mária Mareková, Miroslava Rabajdová

**Affiliations:** 1 Department of Medical and Clinical Biochemistry, Faculty of Medicine, P. J. Šafárik University, Trieda SNP 1, 040 11 Košice, Slovakia; 2 Department of Gynaecology and Obstetrics, Faculty of Medicine, P. J. Šafárik University, Trieda SNP 1, 040 11 Košice, Slovakia; 3 Center for Assisted Reproduction ­ Gyncare, Magnezitárska 2/C, 040 13 Košice, Slovakia

**Keywords:** Spent embryo culture media, Secretome, Metabolomics, Fluorescence, Implantation, miRNA

## Abstract

**Purpose:**

In vitro fertilization occurs in a controlled laboratory setting, where oocytes are fertilized by sperm, and the resulting embryos are cultured to the blastocyst stage before transfer to the uterus. The secreted/consumed substances by the embryo in the extracellular environment (secretome) contain a variety of molecules that may provide insights into embryo quality. This study presents new perspectives on the non-invasive and cost-effective assessment and evaluation of embryos during the IVF process, utilizing a spent embryo culture medium (SECM).

**Methods:**

The SECM was used from blastocysts prepared for a single blastocyst transfer and was analyzed in two groups—the SECM with successful (F) (n = 30) and unsuccessful (N) (n = 36) embryo implantation in the woman's uterus. Building on our previous next-generation sequencing results, we decided to validate the expression levels of specific miRNAs, particularly hsa-miR-16-5p and hsa-miR-92a-3p, to assess their potential to predict embryo implantation success.

**Results:**

Our results demonstrate different expression levels of miRNA molecules in the monitored groups, which could lead to their use in non-invasive analysis of the implantation potential of embryos in the IVF process. In this study, we employed a metabolomics approach using 3D fluorescence analysis of SECM to identify differences between the studied groups, F and N. Our preliminary results indicate a slightly increased metabolic activity in the group with unsuccessful embryo implantation group.

**Conclusions:**

This is our pilot study where we demonstrated the use of two approaches in analyzing the SECM to predict the implantation potential of embryos in the IVF process which promises further development.

## What does this study add to the clinical work

**Table:** 

Using fluorescence analysis in combination with microRNA analysis from spent embryo culture media (SECM) as waste material could lead to efficient selection of embryos with high implantation potential for transfer without invasive intervention in clinical practice.	

## Introduction

Infertility, defined as the inability to conceive after one year of unprotected intercourse [1], is influenced by factors such as age, gynecological and andrological issues, hormonal imbalances, and genetic abnormalities [2]. Assisted reproduction techniques, such as in vitro fertilization (IVF), are increasingly used to address fertility issues. For a successful single embryo transfer, the proper selection of an euploid embryo and a receptive endometrium, as well as the determination of the implantation window, are essential [3]. Despite the growing number of IVF transfers, the success rate remains below 35% for women under 35 years and 25% for women over 35 years. It rapidly decreases at age after 40 years [4], presenting a significant challenge. This is why there is a strong emphasis on exploring new possibilities and challenges to improve IVF transfer success rates, with one potential solution being the accurate analysis of spent embryo culture media (SECM).

The culture medium in which the embryo develops should create optimal conditions for its growth, ensuring that its metabolic needs are met while minimizing stress during in vitro cultivation. The embryo culture system includes essential components for proper cell development, such as water, ions, carbohydrates, amino acids, macromolecules, vitamins, antibiotics, chelating agents, nucleic acid precursors, hormones and growth factors [5]. The embryo is cultivated in the medium from day one until it reaches the blastocyst stage, which occurs on the fifth or sixth day (late blastocyst) after fertilization. At this point, the blastocyst is suitable for transfer to the endometrium for implantation [6]. During its pre-implantation development, the embryo can consume certain components but also secrete various substances into the extracellular environment.

The culture medium provides the extracellular environment for early embryo development during IVF cultivation. In reproductive medicine, the quality of the culture medium is critical, as it is one of the most important factors influencing the success of infertility treatment [5]. An embryo developing in the culture medium can reflect its condition by secreting molecules such as proteins, metabolites, transcripts, small RNAs, DNAs, and extracellular vesicles into the medium. This set of molecules secreted by the embryo into its extracellular environment is known as the embryo secretome [7]. Numerous studies have explored the analysis of proteomics [8, 9], metabolomics [10–12], transcriptomics [13, 14], and extracellular vesicles [15, 16] concerning embryo quality and implantation potential, with particular emphasis on the small non-coding RNAs (sncRNAs) present in the SECM.

As mentioned above, among all the analyzed molecules from SECM, sncRNAs are the most studied in determining the potential of a high-quality embryo or an embryo with a high implantation potential. RNA molecules play diverse roles in cells beyond mRNA, which carries the genetic information required for protein synthesis. In contrast, non-coding RNA (ncRNA) molecules are essential for regulating gene expression and safeguarding the genome against viruses and transposable elements [17]. In recent years, research has underscored the role of sncRNAs, including microRNAs (miRNAs), small interfering RNAs (siRNAs), piwi-interacting RNAs (piRNAs) and tRNA-derived small RNAs (tsRNAs) in the reproductive system concerning embryo quality or implantation success in the IVF process [18–21]. RNA molecules like miRNA [21–24] and piRNA [25, 26] are the most extensively studied potential biomarkers for embryo quality and implantation in SECM, while information on other transcripts remains limited.

Metabolomics involves analyzing all metabolites secreted and consumed by the embryo into the culture medium [10]. It is well established that during development in the culture medium, the embryo consumes certain amino acids for growth while releasing other amino acids [27, 28]. These biochemical intermediates, which fluctuate in response to metabolic and environmental changes, provide valuable insights into cellular activity and can serve as potential biomarkers for embryo viability or implantation potential [11, 29].

An ideal embryonic biomarker should be stable, embryo-specific and easily detectable while the methods used for analysis should be preferably non-invasive [22]. Analyzing the SECM secretome could identify key parameters for successful IVF outcomes. Moreover, SECM analysis is a non-invasive, cost-effective and objective method compared to current practices, such as invasive embryo interventions, genetic testing, and subjective embryo morphology evaluation [30].

The gold standard for analyzing sncRNAs, particularly miRNAs, is next-generation sequencing (NGS). NGS allows massive parallel sequencing of large data sets, which, after thorough bioinformatic analysis, yields information about the expression of individual miRNA molecules detected in SECM [31]. While this approach may seem financially demanding, quantitative real-time PCR (qRT-PCR) can validate the relative expression of specific miRNA molecules from SECM, especially when analyzing a smaller number of target miRNAs. This method is generally regarded as more cost-effective than NGS.

Profiling the metabolome of the SECM requires highly sensitive tools that can detect even subtle changes in its composition [18]. Recently, various spectroscopy techniques, including Raman spectroscopy, nuclear magnetic resonance (NMR) spectroscopy, IR spectroscopy, gas chromatography coupled with mass spectrometry (GC–MS) and high-performance liquid chromatography followed by mass spectrometry (HPLC–MS), have been employed to investigate the full embryonic metabolome [12]. However, the mentioned methods require more demanding sample preparation, expensive equipment, long-term measurements and analysis of a large amount of data. In contrast, spectral methods like fluorescence spectrophotometry offer a straightforward, rapid, and cost-effective way to analyze the metabolome. In comparison to other spectrophotometric methods, fluorescence spectrophotometry provides sensitive detection with excellent selectivity, making it widely applicable in medical research [32]. Our previous studies analyzing various biological materials, including serum, urine, cerebrospinal fluid, tissues and tears [33–35], have identified differences in metabolites across patient groups. Consequently, we aimed to explore these differences in a less commonly studied biological material, such as SECM.

## Materials and methods

### Patients and biological material

Biological materials were collected during standard diagnostic procedures and examinations at the Center for Assisted Reproduction Gyncare in Košice, Slovakia. The patients were informed about the use of the samples and signed the informed consent of the participant in biomedical research. The collection and analysis of the samples followed the requirements of the Ethics Committee of the Faculty of Medicine. From the 1113 patients who participated in the study, a subgroup of 73 patients with idiopathic infertility, aged 18–37, was selected. All participating patients had not previously undergone IVF therapy and had no previous IVF pregnancies. The oocytes collected from these patients underwent in vitro fertilization and the embryos were cultured to the blastocyst stage, i.e. on the 4th/5th day (D4/D5) after fertilization in G-TL™ culture medium (Vitrolife). Spent embryo culture medium (SECM) was obtained from blastocysts prepared for a single embryo transfer. SECM with a volume of about 20 µl was collected during the IVF process on the day of embryo transfer to the uterus (on the 4 h/5th day after fertilization) and stored at −80 °C. Of the total samples analyzed (*n* = 73), 49% (*n* = 35) were from successfully implanted blastocysts, while 51% (*n* = 37) were from non-successfully implanted blastocysts. The successful implantation after IVF treatment was confirmed by measuring serum Human chorionic gonadotropin (hCG) levels (above 10 IU/L) and performing an ultrasound examination on the 10th day post-embryo transfer.

### Analysis of relative expression of hsa-miR-16-5p and hsa-miR-92a-3p

MiRNAs were isolated from SECM samples using the commercial miRNeasy Micro Kit (Qiagen) following the manufacturer's guidelines. The miRNA isolation protocol was slightly adjusted due to a smaller volume of available biological material (SECM) and included an additional DNase treatment step. Before the isolation process, the samples were categorized based on the result of embryo implantation success – successful (F) or unsuccessful (N) implantation, according to the day of transfer – day 4 (4D) or day 5 (5D), specifically into 4D F (*n* = 6), 5D F (*n* = 24), 4D N (*n* = 7) and 5D N (*n* = 29) groups. The culture medium not used for embryo culture constituted the control group M (*n* = 3). A 60 µl pool was created from each particular group, and miRNAs were isolated. The concentration and purity of the isolated miRNA were determined using a NanoDrop 2000 C Spectrophotometer. After isolating miRNA molecules from the pooled SECM groups, the miRNAs were diluted to a final concentration of 2 ng/µL. Following this, complementary DNA (cDNA) was synthesized from the miRNAs using the TaqMan™ MicroRNA Reverse Transcription Kit (Applied Biosystems), following the manufacturer's instructions. This was followed by qPCR analysis of selected miRNA molecules, namely hsa-miR-16-5p and hsa-miR-92a-3p using TaqMan® Universal Master Mix II, no UNG (Applied Biosystems) according to the manufacturer's instructions.

### Fluorescence analysis of SECM

SECM samples were processed using three-dimensional fluorescence analysis. The fluorescence method can reveal any changes at the level of the metabolome in the investigated biological material, by determining characteristic features, i.e. fluorophores or groups of fluorophores that distinguish individual groups from each other. A total volume of 1 μL of sample was 2500 times diluted in PBS solution as well as in PBS:DMSO solution (1:1). Fluorescence spectra were recorded on a Perkin-Elmer LS 55 luminescence spectrophotometer in a 0.5 cm cuvette at room temperature. The slit was set to 5 nm for both excitation and emission wavelengths with the wavelength scanning speed of 1200 nm/min. Synchronous fluorescence spectra were measured in the excitation interval from 200 to 400 nm with a wavelength difference Δ_λ_ = 30–170 nm and were used to create a contour map, the so-called fluorescent fingerprint. For easier comparison of the individual samples, 3D synchronous spectra were further processed into fluorescence profiles, as described in our recent publication [35].

### Statistical analysis

Statistical analysis of gene expression for selected miRNA molecules was conducted using the GraphPad Prism program (version 8) with a two-tailed unpaired t-test. Probability values of *p < 0.05 were deemed statistically significant. Data are expressed as means ± standard error of the mean (SEM), with expression level analysis performed in technical duplicates. The statistical processing of fluorescence spectrophotometry was also carried out using both Origin and GraphPad Prism software, following the same approach used for gene expression analysis. Outliers were identified and removed for each dataset using the ROUT method (Q = 1%). Normality was assessed using the Shapiro–Wilk test, and since the fluorescence intensity data exhibited a non-normal distribution, the Mann–Whitney U test was used to compare fluorescence intensities between the fertile and non-fertile SECM groups. Given the non-normal distribution of fluorescence intensities, bar graphs with medians and interquartile ranges (IQRs) were used for visualization instead of mean-based representations. Statistical significance was determined using a two-tailed approach, with significance levels interpreted as *p* < 0.05 (*), p < 0.01 (**), and p < 0.0001 (***).

## Results

### Analysis of miRNAs from SECM – molecular approach

**Fig. 1:**
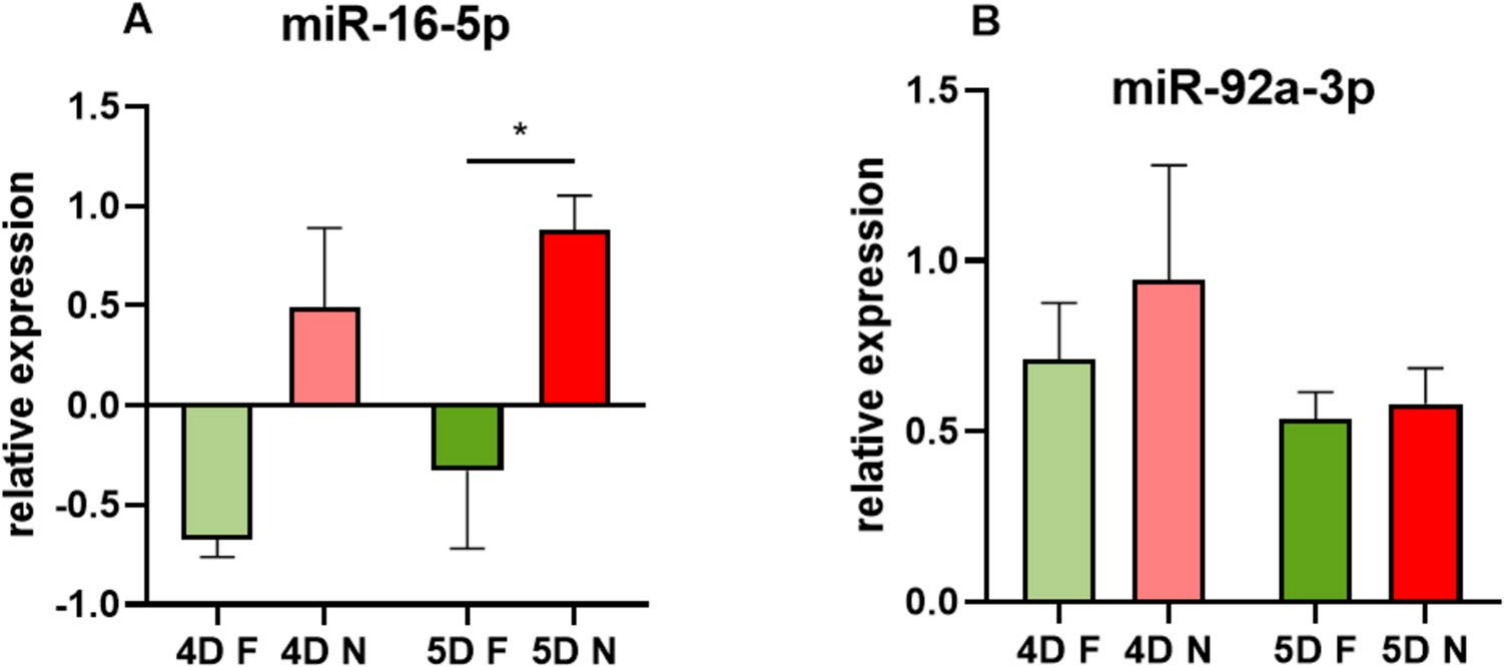
Relative expression of miR-16-5p (**A**) and 92a-3p (**B**) in SECM of 4D/5D embryos in the group with successful (F) and unsuccessful embryo implantation (N). Values are normalized to the free culture medium (M) (*p < 0.05). Error bars show SEM

The first miRNA analyzed from SECM using qRT-PCR was hsa-miR-16-5p, as shown in Fig. 1A and Table 1. In the 4D N group of unsuccessfully implanted blastocysts, its expression level increased by 230% compared to the 4D F group of successfully implanted blastocysts in 4-day embryos. A significant difference (*p = 0.030) was observed for the 5-day embryo (5D) group of unsuccessfully implanted blastocysts (5D N), where the expression of miR-16-5p was increased by 137% compared to the successfully implanted blastocysts (5D F) group.

**Table 1:** Relative expression of miR-16-5p and miR-92a-3p in SECM normalized to free culture medium. Values are shown as means ± SEM

	4D F	4D N	5D F	5D N
miR-16-5p				
Relative expression	−0.671 ± 0.091	0.490 ± 0.400	−0.328 ± 0.675	0.881 ± 0.299
* p*-value	0.106		0.030*	
miR-92a-3p				
Relative expression	0.710 ± 0.166	0.944 ± 0.336	0.536 ± 0.138	0.581 ± 0.182
* p*-value	0.596		0.745	

Values of *p < 0.05 were considered statistically significant

Differences between 4 and 5D embryos in miR-16-5p expression can be observed, with an increased expression level of 104.6% in the group of successfully implanted 5D F embryos compared to 4D F (*p* = 0.590). In the group of unsuccessfully implanted blastocysts, slight differences in expression can be observed. In contrast, for 5D N the level of expression was increased by 44.4% compared to 4D N, but without a statistical significance (*p* = 0.334). For miR-16-5p, a higher expression was observed in the free culture medium (M) compared to SECM embryos with successful implantation, therefore negative expression values can be observed as they were normalized to the free culture medium (M).

The expression level of miR-92a-3p is increased by 24.5% in the group of SECM samples of unsuccessfully implanted embryos (4D N) compared to successfully implanted embryos (4D F), as is shown in Fig. 1B as well as in Table 1. In comparison, a slightly increased relative expression (7.8%) was observed in the group of 5-day embryos for unsuccessfully implanted blastocysts (5D N) (*p* = 0.745) compared to successfully implanted ones (5D F).

Differences in the expression of miR-92a-3p can also be observed when comparing the SECM of a 4-day-old embryo (4D) with the SECM of a 5-day-old embryo (5D). A slightly higher relative expression of miR-92a-3p was observed in the 4-day-old embryo group 4D F versus 5D F by 23% and by 38.5% for 4D N versus 5D N. However, all these observed differences were not statistically significant, in the F group with a value of *p* = 0.330 and in group N with a value of *p* = 0.234.

#### Fluorescence analysis of IVF culture medium – metabolomic approach

**Fig. 2:**
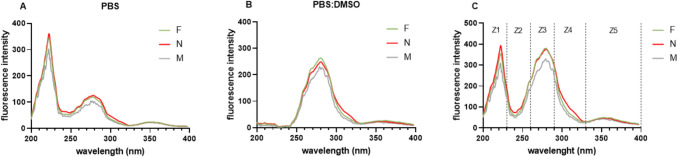
Fluorescence profiles **A**. Fluorescence profiles of average SECM spectra of samples in PBS. The fluorescence profile of a medium diluted with PBS (1: 2500) features two fluorescence emission maxima at 223 and 280 nm, which are attributable to tyrosine and tryptophan, respectively. **B**. Fluorescence profiles of average SECM spectra of samples in PBS:DMSO. **C**. The additive profile represents the average values of all samples in given groups with division into zones 1–5. The curves represent the maximum values in individual groups. F, SECM of successfully implanted blastocyst; N, SECM of unsuccessfully implanted blastocyst, M, free culture medium

Because of the limited amount of the culture media available, the fluorescence metabolomic analysis was mainly focused on the UV range of the fluorescence spectra, where the aromatic amino acids (Tryptophan, Tyrosine and Phenylalanine)—either as free or as part of proteins and their metabolites can be detected. A straightforward evaluation of the metabolic activity of the embryos from successful and unsuccessful implantations is difficult due to the high background fluorescence caused by the presence of albumin and essential amino acids in the medium. The fluorescence profile of a medium diluted with PBS (1: 2500) features two fluorescence emission maxima, located at 223 and 280 nm attributable to tyrosine and tryptophan, respectively (Fig. 2A), whereas the profile of a medium diluted in a mixture of PBS and DMSO (1: 2500) which shows only the more pronounced fluorescence emission of tryptophan (Fig. 2B).

**Fig. 3:**
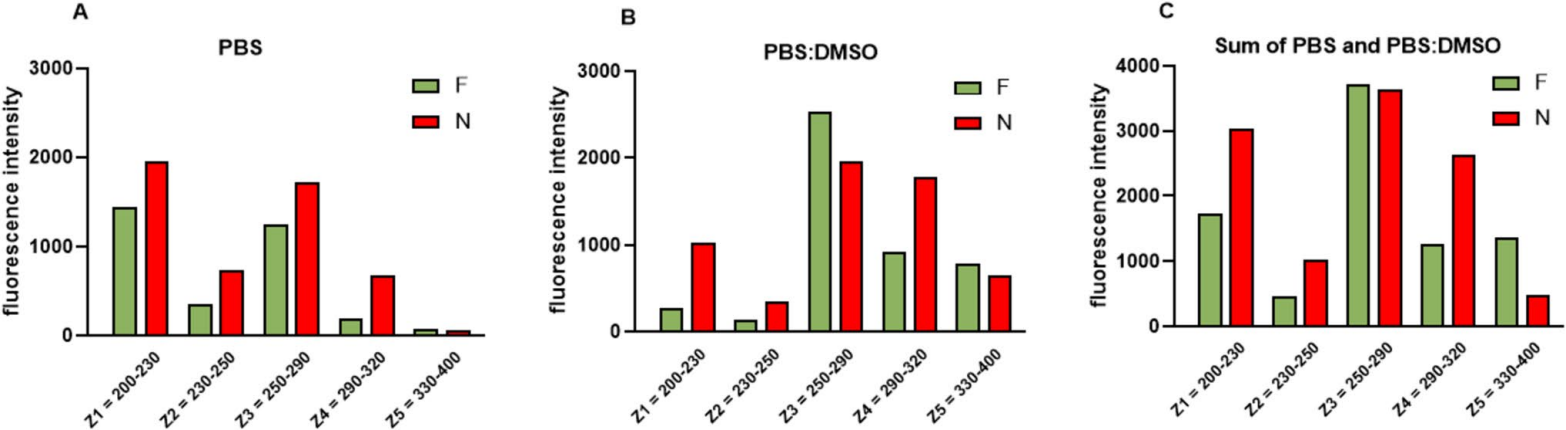
Display of individual zones for given groups (F/N). **A**. Fluorescence of zones after subtraction of free culture medium in PBS. **B**. Fluorescence of zones after subtraction of free culture medium (M) in PBS:DMSO. **C**. Sum of respective zones in PBS and PBS:DMSO. The values given are the average of all samples. F, SECM of successfully implanted blastocyst; N, SECM of unsuccessfully implanted blastocyst

**Fig. 4:**
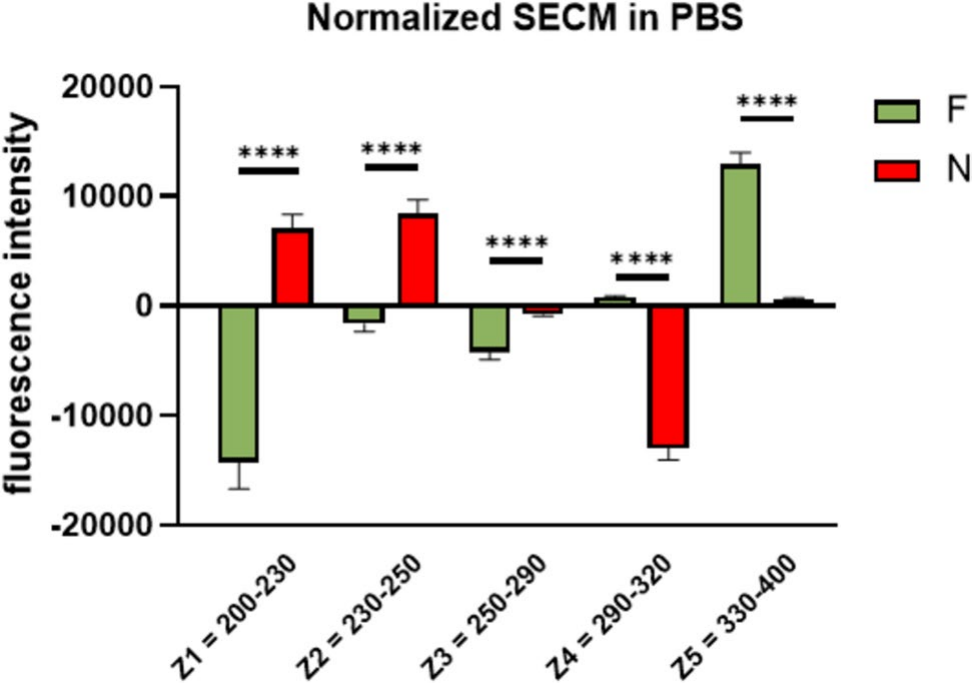
Statistical analysis of the individual zones normalized to free culture medium with *p* < 0.0001 (****). F, SECM of successfully implanted blastocyst; N, SECM of unsuccessfully implanted blastocyst

For the complex evaluation of the metabolic activity of the tested culture media, an additive profile of the sample was designed (Fig. 2C). The additive profile represents a novel approach that describes the total fluorescence of the culture medium measured in both dilutions (PBS and PBS:DMSO) in a certain region of the spectrum and thus allows a more thorough comparison of the metabolic activity. While this strategy has been used on other biological materials such as serum, urine or tear fluid, best to our knowledge it has not been applied in the analysis of culture media. Based on the distribution of the fluorescence peaks and the observed differences in the profiles, the additive profile was divided into 5 zones; Z1 = 200–230 nm; Z2 = 230–260 nm; Z3 = 260–290 nm; Z4 = 290–330 nm and Z5 = 330–400 nm (Fig. 2C). The additive profile clearly shows a significantly higher metabolic activity of the embryos from the unsuccessful implantations (N) which is most pronounced in the Z1, Z2 and Z4 zones (Fig. 3). Although the aim of this study was only to provide an initial evaluation of the concept of the division of the fluorescence profile into the zones, a pilot statistical analysis has also been performed. The analysis of the zones normalized to the free medium for individual samples has confirmed the abovementioned opposite trends illustrated by statistically significant differences in the zones Z1, Z2 and Z4 (Fig. 4). Nonetheless, a relatively small number of samples used for the analysis has to be considered as a limitation of the presented statistical analysis.

**Fig. 5:**
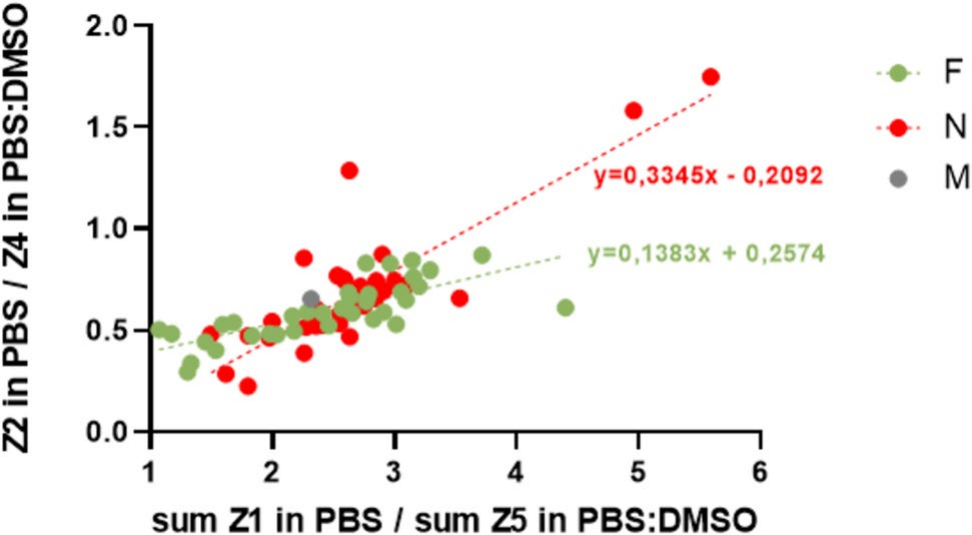
Correlation graph of the dependence of the ratios of individual zones for the given groups. F, SECM of successfully implanted blastocyst; N, SECM of unsuccessfully implanted blastocyst; M, free culture medium

Calculation of the ratios between the individual zones in the respective solvents and their subsequent correlations can be used to design advanced parameters. This concept can be illustrated by the correlation of the ratio of the intermediate zones (Z2 in PBS)/(Z4 in PBS:DMSO) and the ratio of the border zones (Z1 in PBS)/(Z5 in PBS:DMSO) for all analyzed samples (Fig. 5). The trend lines of this correlation for F and N samples show a relative separation which allows their potential differentiation.

To more thoroughly investigate the composition of the SECM it is necessary to distinguish the fluorescence of the albumin (HSA) present in the culture medium from the fluorescence of other proteins and metabolites of aromatic amino acids. The maximum intensity value located in the Z3 zone (λ_EX_ = 280 nm) can be considered as the sum of albumin, other proteins, free tryptophan and its metabolites. The fluorescence of HSA has been determined based on photometrical determination of its concentration in the SECM. Therefore, subtraction of the calculated fluorescence of the HSA provides information about the fluorescence of the remaining metabolites. In addition, when the value of the fluorescence of the remaining metabolites in the free culture medium (M) which can be considered as background fluorescence is further subtracted from the values for SECM from successful (F) and unsuccessful (N) implantations, we can calculate the amount of fluorescence attributable to the metabolic activity of the respective embryos (Table 2). The subsequent comparison of the metabolic activity of F and N samples clearly illustrates the abovementioned higher activity of the embryos from the unsuccessful implantations documented by a ratio of 1.66 (N/F). It is worth noting that this ratio is based on average total fluorescence values and it has not been statistically analyzed. Therefore, a verification of the relevance of this parameter by statistical evaluation on a larger number of samples is required.

**Table 2:** Comparison of fluorescence intensities for total fluorescence, HSA, metabolites and embryo metabolic activity for individual groups

	F	N	M
Total fluorescence	301,582.6	312,698.6	259,570.1
HSA	44,842.3	44,927.5	19,635.0
Metabolites	256,740.3	267,771.1	239,935.1
Metabolic activity of embryos	16,805.2	27,836.0	–

F, SECM of successfully implanted blastocyst; N, SECM of unsuccessful implanted blastocyst; M, free culture medium

## Discussion

Based on the previous results of the NGS analysis [26], we decided to verify the expression of the relevant miRNA molecules, which were significant in predicting implantation success. Hsa-miR-16-5p was present in the inner cell mass (ICM), trophectoderm (TE), and pooled SECM samples of both successfully implanted and unsuccessfully implanted blastocysts [23], as well as in the placenta [36]. MiR-16-5p is expressed in both human and mouse endometrium, targeting the angiogenic signaling pathway [37]. Using qPCR analysis, an increased expression level for miR-16-5p was identified in the follicular fluid of high-quality 3-day embryos [38]. The target genes of miR-16-5p include some important genes in the implantation process, such as nuclear factor kappa B subunit 1 (*NFKB1),* vascular endothelial growth factor A *(VEGFA),* AKT serine/threonine kinase *(AKT)* and SRY-box transcription factor 2/4 (*SOX2/4)*. Pro-inflammatory transcription factor NF-κB is important during pregnancy, especially during embryo implantation [39]. In addition, increased expression of this miRNA has been observed in pregnant women with polycystic ovary syndrome [40], in the plasma of women associated with restricted fetal growth [41], preeclampsia [42] or studied in connection with ovarian cancer [43]. Exiqon analysis (whole human miRNome qPCR analysis) of SECM samples showed a higher level of miR-16-5p (along with miR-371a-5p, miR-372-3p and miR-192-5p) in SECM samples of embryos that were successfully implanted, compared to unsuccessfully implanted ones [23], which is opposite to our results. In addition to vascularization of the endometrium, *VEGF-A* also plays a role in promoting embryo implantation, improving endometrial receptivity as well as communication between the embryo and the endometrium [44], and *AKT* is essential during early embryonic development [45]. The transcription factors *SOX2* and *SOX4* are potential target genes of miR-16-5p. *SOX4* is an important regulator of human endometrial decidualization and its disruption is associated with recurrent implantation failure during the IVF process [46] and *SOX2* has a role in blastomere fate in favor of ICM formation [47]. Another potential effect of miR-16-5p in the processes of pre-implantation development of the embryo as well as implantation is shown in Table 3.

**Table 3:** Probable impact of miR-16-5p and miR-92a-3p from SECM in the IVF process (↓ – downregulation, ↑ – upregulation)

miRNA	Target gene	Impact of miRNA	Source
miR-16-5p	↓ *NFKB1*	↓ Embryo implantation	[39]
	↓ *AKT*	Disruption of the early development of the embryo	[45]
	↓ *VEGFA*	↓ Endometrial receptivity, ↓ Embryo implantation, disturbed interaction between embryo and endometrium ↓ Angiogenesis	[44, 57]
	↓ *SOX2*	↓ Formation of ICM during embryo development	[47]
	↓ *SOX4*	↓ Decidualization	[46]
miR-92-3p	↓ *SOX4*	↓ Decidualization	[46]
	↓ *BTG2*	↑ Embryonic development	[56]
	*TOB1*	Embryonic dorsoventral patterning	[55]
	↓ *WNT5 A*	↓ Decidualization	[58, 59]
	↓ *MDM2*	↓ Adhesion of the blastocyst to the endometrium	[54]
	↓ *PTEN*	↑ Proliferation of stromal cells, decidualization	[60, 61]
	↓ *SOCS1*	↑ Anti-apoptotic processes	[62]

In this paper, the expression level of miR-16-5p was significantly upregulated in the SECM group with an unsuccessfully implanted embryo from day 5 after fertilization. Potential target genes of miR-16-5p suggest its role in suppressing processes important in preimplantation embryo development as well as during implantation and subsequent decidualization.

By qPCR analysis from SECM, a slightly increased level of miR-92a-3p was observed in the SECM group with unsuccessfully implanted embryos compared to the successfully implanted group. Several studies have dealt with the analysis of miR-92a-3p expression level precisely in connection with gynecological disorders [9, 48], potential determination of embryo quality from embryo culture medium [49] and blastocoel fluid [50].

Communication within the cumulus-oocyte complex (COC) is crucial in acquiring the developmental competence of the oocyte during folliculogenesis as well as in oocyte maturation [51]. The study by Dell'Aversana et al. [52] investigated the molecular and regulatory mechanisms mediated by miRNAs in COCs that affect the regulation of oocyte competence in women undergoing IVF. MiR-92a-3p together with miR-16-5p were upregulated in COCs in association with younger women's age (≤ 35 years) compared to lower expression in the group of older women (≥ 36 years) undergoing IVF [52]. The level of miR-92a-3p has also been studied in gynecological disorders such as endometriosis [9] and polycystic ovary syndrome (PCOS) [48], where its expression level was reduced. Endometriosis, as well as PCOS, is a well-known cause of infertility that slows down the implantation process as well as reduces the receptivity of the endometrium [53]. The target gene for miR-92a-3p is the proto-oncogene gene mouse double minute homologue 2 (*MDM2)* with its role in contributing to blastocyst adhesion to the endometrium [54] but also supporting endometriosis [9]. MiR-92a has an essential role in blastocyst development as well as implantation potential, and its increased expression was correlated with developing embryos based on microarray analysis [49], which is in negative correlation to our findings. For a better understanding of the early embryonic development of the embryo as well as its quality, the authors Battaglia et al. [50] investigated the presence of miRNA molecules in blastocoel fluid. Using microarray analysis, they identified several molecules present in blastocoel fluid, including miR-92a-3p. In Table 3, potential target genes with their effect within the human embryo are listed. Target genes of miR-92a-3p according to the DIANA predictor include the *SOX4* gene, which is important in the process of decidualization [46]. Some anti-proliferative genes, such as B-cell translocation gene 2 (*BTG2*) and transducer of ERBB2-1 (*TOB1*) were identified as target genes of miR-92a-3p. *BTG2* and *TOB1* could have a role in early embryonic development and growth regulation [55, 56].

Small non-coding RNAs secreted by the embryo into the culture medium mediate essential communication between the blastocyst and the endometrium. Studies confirm that the miRNAs present in the culture medium come from the embryo at the blastocyst stage [22]. The sncRNA secretome has a unique role in blastocyst–endometrium communication, as confirmed by the distinct expression profile of sncRNAs from trophectoderm and ICM cells [23].

The diagnostic potential of individual small non-coding RNAs from the culture medium of the blastocyst is not significant considering that the expression of sncRNAs was different according to several studies and individual sncRNAs can regulate several target molecules. However, a set of molecules sncRNAs could represent a sensitive predictive tool for distinguishing high-quality and low-quality embryos for transfer to the uterus during the IVF process, for example, in combination with the analysis of metabolome of some major proteins involved in the pathways regulated by the respective sncRNAs.

The gold standard of traditional metabolomic analysis is chromatographic separation followed by the identification of individual metabolites by mass spectrometry (MS) or NMR. ^1^H NMR metabolomic profiling of embryo culture media has been used to identify biomarkers associated with embryo reproductive potential [63], whereas Eldarov et al. [12] analyzed metabolomic profiles of SECM of human embryos with different morphology and karyotype using HPLC–MS. Spectral methods are even more promising, especially for their easy integration into routine clinical diagnostics. Their results are in the form of a spectral metabolomic signature that can be used to distinguish embryo quality. Raman spectroscopy [63], Fourier transform infrared spectroscopy (FTIR) [64], near-infrared (NIR) spectroscopy [65] were all already used to assess embryo viability based on spectral metabolomic profiling of the SECM.

However, fluorescence analysis, to the best of our knowledge, has not been applied to SECM monitoring. The paper presents a pilot study of the application of synchronous spectra to SCEM in an original way. The previous experience with the application of synchronous spectra to distinguish biological material samples from endometrial cancer patients from the control group served as an inspiration for the application of 3D synchronous spectra to SCEM analysis. The selection of specific spectral characteristics from the blood serum of gynecological patients was successfully used to identify patients with endometrial cancer compared to healthy subjects. Visual differences were confirmed by machine learning models [66]. Fluorescence metabolome of urine presented as a synchronous spectrum divided into spectral zones also showed spectral differences between patients with endometrial cancer, control and benign groups [67]. Therefore, the division of the fluorescence profile into zones has been inspired by the abovementioned studies.

The results of the fluorescence analysis of SECM revealed a detectable difference between the metabolic activity of the embryos from the successful and unsuccessful implantations. Previous studies focused on the metabolome have indicated changes in the metabolism of embryos before implantation, particularly by examining amino acid, glucose, and pyruvate metabolism [10]. While the metabolic signature of SECM has been suggested as an effective method for assessing embryo viability and developmental potential [68], the amino acid "fingerprint" from the culture medium could serve as a valuable indicator of the embryo's implantation potential [11]. Although numerous studies have investigated amino acids including glutamate, glutamine, serine, alanine or methionine [69, 70], the aromatic amino acids that contribute to the fluorescence of SECM haven’t been evaluated. Therefore, it is not possible to clarify the observed fluorescence differences based on concurrent studies. Nevertheless, the presented pilot study holds promise for further investigation and the combination of 3D synchronous spectra with machine learning tools can lead to improvement of embryo selection in the IVF process.

## Conclusions

The determination of the expression for the selected miRNAs (miR-16-5p and miR-92-3p) has brought some interesting results. A relatively large difference in the expression of the miR-16-5p in the SECM of the embryos from the successful and unsuccessful implantation suggests that these molecules might play an important role in embryonal development. Our study also illustrates the importance of the timeframe, since differences in the expression of both miRNAs between the embryos implanted on the 4 th or 5 th day were detected. As mentioned above, a potential miRNA-based evaluation would require a broader panel of different sncRNAs for a reliable diagnostic.

The fluorescence analysis of the SECM represents an alternative approach to the non-invasive investigation and selection of the embryos suitable for implantation. An exhaustive analysis of the obtained fluorescence data revealed that the metabolic activity calculated by comparing free culture media to the SECM from both studied groups F and N seem to be a promising marker. However, to the best of our knowledge, there is no complex metabolomic study of the SECM focused on the proteins, aromatic amino acids and their metabolites, and therefore it is currently impossible to provide a definite explanation of the observed differences. Furthermore, a validation of this preliminary study on a larger number of samples is necessary.

In summary, this work aimed to outline novel perspectives in the non-invasive and inexpensive investigation and evaluation of embryos for the IVF process based on the SECM. Even though the results presented herein are to be considered only as a pilot study, both the sncRNA analysis and the fluorescence metabolome analysis appear to be interesting for further development.

## Data Availability

No datasets were generated or analysed during the current study.

## Abbreviations

AKT: AKT serine/threonine kinase;
BTG2: B-cell translocation gene 2
COC: Cumulus-oocyte complex
F: SECM of successfully implanted blastocyst
FTIR: Fourier transform infrared spectroscopy
GC–MS: Gas chromatography coupled with mass spectrometry
hCG: Human chorionic gonadotropin
HPLC–MS: High-performance liquid chromatography followed by mass spectrometry
ICM: Inner cell mass
IVF: In vitro Fertilization
M: Free culture medium
MDM2: Mouse double minute homologue 2
miRNAs: MicroRNAs
N: SECM of unsuccessfully implanted blastocyst
ncRNA: Non-coding RNA
NFKB1: Nuclear factor kappa B subunit 1
NGS: Next-generation sequencing
NIR: Near infrared
PCOS: Polycystic ovary syndrome
piRNAs: Piwi-interacting RNAs
PTEN: Phosphatase and tensin homolog
qRT-PCR: Quantitative real-time PCR
SECM: Spent embryo culture medium
siRNAs: Small interfering RNAs
sncRNAs: Small non-coding RNAs
SOCS1: Suppressor of cytokine signaling 1
SOX2/4: SRY-box transcription factor 2/4
TE: Trophectoderm
TOB1: Transducer of ERBB2-1
tsRNAs: TRNA-derived small RNAs
VEGFA: Vascular endothelial growth factor A
WNT5 A: Wnt family member 5A
